# Importance of carbohydrate antigen (CA 19-9) and carcinoembrionic antigen (CEA) in the prognosis of patients with duodenal adenocarcinoma: a retrospective single-institution cohort study

**DOI:** 10.18632/oncotarget.28406

**Published:** 2023-04-15

**Authors:** Ellery Altshuler, Raymond Richhart, William King, Mahmoud Aryan, Akash Mathavan, Akshay Mathavan, Keegan Hones, Daniel F. Leach, Logan Pucci, Joshua Riklan, Pat Haley, Ilyas Sahin, Brian Ramnaraign, Sherise Rogers, Ibrahim Nassour, Steven Hughes, Thomas J. George, Jesus Fabregas

**Affiliations:** ^1^Department of Internal Medicine, University of Florida, Gainesville, FL 32610, USA; ^2^Department of Internal Medicine, University of Alabama at Birmingham, Birmingham, AL 35294, USA; ^3^University of Florida College of Medicine, Gainesville, FL 32610, USA; ^4^Division of Hematology and Oncology, Department of Medicine, University of Florida, Gainesville, FL 32610, USA; ^5^University of Florida Health Cancer Center, Gainesville, FL 32610, USA; ^6^Division of Surgical Oncology, Department of Surgery, University of Florida, Gainesville, FL 32610, USA; ^7^Department of Radiation Oncology, University of Florida Health, Gainesville, FL 32601, USA

**Keywords:** duodenal adenocarcinoma, carbohydrate antigen, CA 19-9, carcinoembrionic antigen, CEA

## Abstract

Background: Duodenal adenocarcinoma (DA) is a rare malignancy without validated tumor markers. In practice, carcinoembryonic antigen (CEA) and carbohydrate antigen (CA 19-9) are often used in the management of DA, though their prognostic value is unknown.

Materials and Methods: A single-institution retrospective review included patients diagnosed with biopsy-confirmed adenocarcinoma of the duodenum between 2006 and 2021. Peri-ampullary tumors were excluded. Levels of CA 19-9 and CEA were collected as continuous variables and were analyzed as binary variables: normal vs. high, using the maximum normal value as a cut-off (normal Ca 19-9 <35 U/ml; CEA <3 ng/ml). Survival analysis was conducted using Kaplan Meier curves, log-rank test and Cox proportional hazards model.

Results: There were 68 patients included in the final analysis. Median age was 67 years old and median follow-up time was 22.2 months. CA 19-9 and CEA were elevated in 36.8% and 48.5% of patients, respectively. A concomitant elevation of both tumor markers was associated with worsened OS (HR 2.140, 95% CI: 1.114–4.112; *p* = 0.019). After controlling for age and sex on multivariate analysis, elevation in both CA 19-9 ≥35 and CEA ≥3.0 remained significantly associated with increased mortality (HR 2.278, 95% CI: 1.162–4.466; *p* = 0.016).

Conclusions: In summary, CA 19-9 and, to a lesser extent, CEA, show promise as prognostic markers in DA. Larger studies are needed to validate their use and to evaluate their performance as markers of recurrence.

## INTRODUCTION

Duodenal adenocarcinoma (DA) accounts for 0.5% of all gastrointestinal cancers [1]. With an annual incidence of 3.7–5.4 cases per million, DA makes up between a third and a half of small bowel adenocarcinomas [2, 3]. Surgery remains the gold standard for curative intent, with observational data suggesting chemotherapy and radiotherapy as reasonable adjuvant modalities [4]. Despite these treatment modalities, DA portends a poor prognosis [2].

Although tumor markers are employed in the management of DA to inform prognosis and to evaluate disease response to treatment, their prognostic value is unknown [2]. Carcinoembryonic antigen (CEA) and carbohydrate antigen (Ca 19-9) are widely used in colon and pancreatic adenocarcinoma, respectively. However, it is not known if CEA and CA 19-9 are predictive or prognostic in patients with DA [5]. It is also not known which one of these two markers is more important in predicting overall survival (OS). Both CEA and CA 19-9 are commonly used in the monitoring of duodenal adenocarcinoma despite a lack of evidence supporting this practice. CEA is commonly used, with the rationale being that DA resembles the course of patients with colon cancer. Meanwhile, CA 19-9 levels are obtained with the justification that DA more closely resembles pancreatobiliary malignancies.

To our knowledge, there are no studies evaluating the prognostic importance of CEA and Ca 19-9 in patients with DA. We aimed to answer the question of whether these tumor markers are associated with clinical outcomes in patients with DA. We used a retrospective institutional database at the University of Florida (UF). We evaluated the association between CEA and Ca 19-9 with overall survival. Our findings provide the practicing clinician with real world information about which one of these markers is more useful in establishing the prognosis for patients with DA.

## RESULTS

### Patient characteristics

Sixty-eight patients were treated for DA at UFHSH over a 16-year period. Baseline characteristics are included in Table 1. Forty patients (59%) were male, and 44 patients (65%) had a history of smoking. Median age was 67 years and median follow-up was 22.2 months. Five tumors (7%) were discovered incidentally, and 63 tumors (92.9%) were discovered as part of a symptom-driven workup. Abdominal pain (34%) was the most common chief complaint upon presentation among symptomatic patients.

**Table 1:** Patient characteristics

Variable	Patients (*n* = 68) number (%)
Age, median (range)	67.0 (22–82)
Sex	
Male	40 (59%)
Female	28 (41%)
Race	
White	51 (75%)
Black/African American	11 (16%)
Hispanic/Latino	2 (3%)
Presenting Complaint	
Abdominal pain	23 (34%)
Blood in stool	14 (21%)
Fatigue	11 (16%)
Jaundice	8 (12%)
Early satiety	12 (18%)
Incidental imaging finding	5 (7%)

### Disease characteristics

Tumor characteristics and disease outcomes are listed in Table 2. The most common location was second part of the duodenum (29%). The diagnosis of DA was most frequently made endoscopically (60%). 37% of patients had metastases at the time of diagnosis.

**Table 2:** Disease characteristics

Variable	Patients (*n* = 68) number (%)
Primary tumor size, cm; median (range)	3.95 (0.3–9.8)
Duodenal location	
First	10 (15%)
Second	20 (29%)
Third	7 (10%)
Fourth	4 (6%)
Stage	
I	6 (9%)
II	11 (16%)
III	13 (19%)
IV	25 (37%)
Histological Diagnosis	
Laparoscopic	22 (32%)
Endoscopic	41 (60%)
Biopsy of metastasis	3 (4%)
Treatment	
Surgical resection	22 (32%)
Chemotherapy	43 (63%)
Radiation	15 (22%)
Survival, months	
Median	11.5
Mean	25.4

### Survival analysis

**Figure 1:**
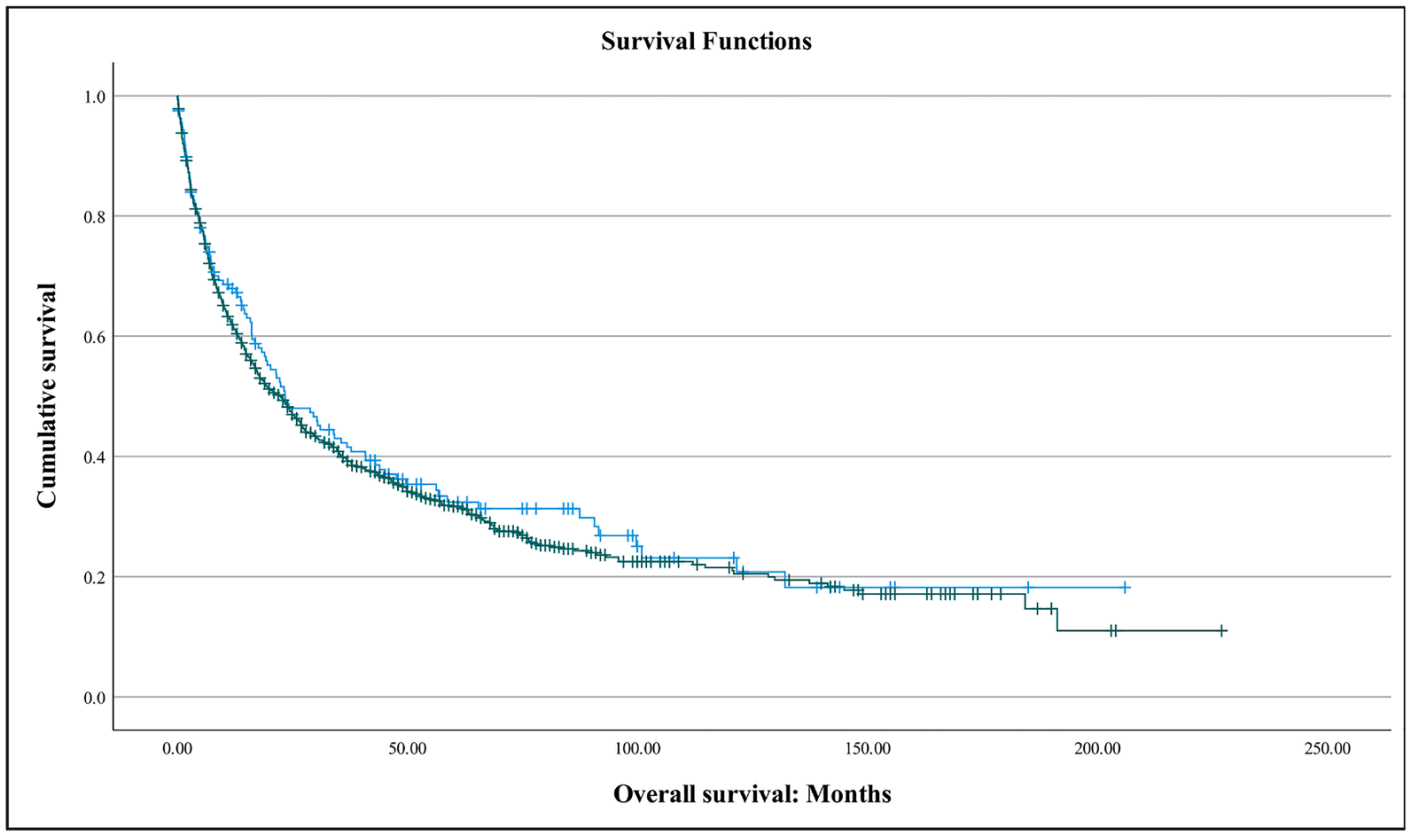
Kaplan-Meier survival curve of duodenal adenocarcinoma patients with elevated CEA (green) and with normal CEA (blue). Patients with an elevated CEA had an estimated median OS of 13.2 months vs. 65.3 months normal levels (*p* = 0.087).

**Figure 2:**
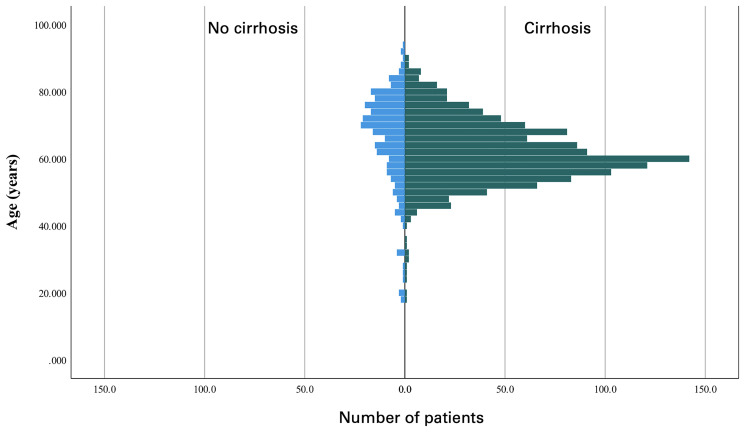
Kaplan-Meier survival curve of duodenal adenocarcinoma patients with elevated CA 19-9 (green) and with normal CEA (blue). Patients with an elevated CA 19-9 had an estimated median overall survival (OS) of 8.4 months vs. 27.2 months normal (*p* = 0.020).

CA 19-9 was elevated in 36.8% of patients, whereas the CEA was elevated in 48.5%. Kaplan-Meier survival functions for these tumor markers are provided below (Figures 1 and 2). Median CA 19-9 was 7.5 U/ML, while median CEA was 2.9 ng/M . Mean CA 19-9 was 1957 U/ML and CEA 13.4 ng/ML. No difference was seen in levels based on race; median CA 19-9 was 12.0 U/ML in white patients and 7.0 U/ML in black patients (*p* = 0.810) while median CEA was 3.4 ng/M in white patients and 9.3 ng/M in black patients (*p* = 0.155) (Table 3). Patients with an elevated CA 19-9 had an estimated median overall survival (OS) of 8.4 months vs. 27.2 months in patients with normal levels (HR 1.971, 95% CI: 1.102–3.524; *p* = 0.022). Patients with an elevated CEA had an estimated median OS of 13.2 months vs. 65.3 months normal levels (HR 1.785, 95% CI: 0.911–3.498; *p* = 0.091). A concomitant elevation of both tumor markers was associated with worsened OS (HR 2.140, 95% CI: 1.114–4.112; *p* = 0.019). On univariate analysis, odds of death were significantly higher with age > 60 (HR 1.861, 95% CI: 1.003–3.453; p = 0.049) and stage IV disease (HR 1.648, 95% CI: 1.145–2.372; *p* = 0.007). Odds of dying were significantly decreased with surgical treatment (HR 0.367, 95% CI: 0.192–0.698; *p* = 0.003). After controlling for age and sex on multivariate analysis, elevation in both CA 19-9 ≥ 35 and CEA ≥ 3.0 remained significantly associated with increased mortality (HR 2.278, 95% CI: 1.162–4.466; *p* = 0.016).

**Table 3:** Univariate analysis

Variable	Univariate analysis HR (95% CI)	*P*
Age > 60	1.861 (1.003–3.453)	0.049
Male sex	1.222 (0.679–2.202)	0.503
Size >5 cm	1.087 (0.512–2.308)	0.828
Stage IV	3.289 (1.699–6.369)	<0.001
Surgery (ref: no)	0.367 (0.192–0.698)	0.003
Chemo (ref: no)	0.974 (0.522–1.817)	0.934
Radiation (ref: no)	1.761 (0.854–3.632)	0.125
CA 19-9 ≥35	1.971 (1.102–3.524)	0.022
CEA ≥3.0	1.785 (0.911–3.498)	0.091
CA 19-9 ≥35 and CEA ≥3.0	2.140 (1.114–4.112)	0.019

## DISCUSSION

In this institutional retrospective cohort study, we reviewed the charts of patients treated for DA at UFHSH between 2006 and 2021. Sixty-eight patients were included in the final analysis.

Elevation of CA 19-9 and CEA levels were each associated with clinically meaningful differences in survival; however, only CA 19-9 was statistically significant. A simultaneous elevation of both CEA and CA 19-9 was associated with worse survival than either marker alone. This result remained significant after controlling for age and sex.

To our knowledge, this is the first study to evaluate the role of tumor markers in patients with DA. In fact, this is the largest single institution study in the US evaluating this disease. Other single-center studies have evaluated clinicopathologic characteristics, treatment patterns and clinical outcomes in DA [6–8]. However, none of them has answered the question of whether tumor markers are predictive of OS. The current study adds to the literature by clarifying that elevation of both, CEA and CA 19-9, is associated with a worse OS in DA cases.

With a median average age of 67 and a male majority of 59%, our cohort was demographically similar to prior analyses of DA, which have reported average ages of 51–65 and slight male majorities of 52–60% [9, 10]. This could be because of months, which is slightly lower than the 18–24 months reported in prior studies [8, 9, 11]. This also could be because of late diagnosis in a rural catchment area. Our cohort was notable for the method by which the tumors were discovered. It was previously believed that DA were often discovered incidentally; however, this is likely only true in certain scenarios [12]. In a review of 205 patients with small bowel adenocarcinomas at 11 Japanese institutions, Sakae and colleagues reported that 38% of patients with duodenal cancer were asymptomatic at the time of diagnosis and that these tumors were incidentally discovered [12]. In Japan, screening for gastric cancer with esophagogastroduodenoscopy (EGD) is routine and it is therefore unsurprising that so many DA would be discovered incidentally [1, 2, 5]. In a review performed by the Mayo Clinic in the USA, meanwhile, just 9% of SBAs were discovered incidentally and, in our cohort, 7% were discovered incidentally [13]. Incidental diagnoses are likely uncommon in countries in which routine screening colonoscopy is not recommended for all adults [11].

Our study had many limitations. First, the study has a small sample size. In retrospective studies we can’t account for all confounders, and the small size limits the implementation of the survival models. However, to our knowledge, this is the US-based study with the largest cohort of patients with duodenal adenocarcinoma. Rare diseases are best initially studied by retrospective cohorts, therefore our study is a valid way of studying DA. Second, the retrospective nature introduces bias in our estimates. It is possible that some of the variables inputs are wrong, and this is inherent to large dataset collections. We did our best to identify outliers. Third, the adjustment for confounders was limited due to low number of events.

Notwithstanding these limitations, our findings demonstrate the association between CEA and CA 19-9 and OS in DA patients. The results are relevant to physicians, as they can use this evidence to routinely request these tests and assist in the interpretation of the results. Our findings are also relevant to patients diagnosed with duodenal adenocarcinoma, who gain further prognostic information about their disease. An elevation in the tumor markers can also help in the surgeon’s decision of whether to do additional search for metastatic disease prior to surgery, with a PET CT for example, in case of marked elevation of tumor markers and normal preoperative CT scans. Or at least, to consider a laparoscopic exploration beforehand given the increased risk of disease spread associated with elevations in both markers.

## MATERIALS AND METHODS

### Design

Patients were identified using the University of Florida Shands Hospital (UFHSH) integrated data repository. This database includes all the patients treated in the University system, with electronic medical records available. We selected the UFHSH because we would have access to the clinical records, including tumor markers levels, clinical characteristics, pathology reports, treatment, and survival. Approval for this study was obtained from the University of Florida Institutional Review Board (IRB 202102705).

### Patient population and data collection

Patients were included if they had biopsy-confirmed adenocarcinoma of the duodenum diagnosed between January 1, 2006, and December 31, 2021. Peri-ampullary tumors were excluded. Stages were determined using the American Joint Committee on Cancer Staging, 8th edition [14]. Baseline demographic, pathologic and clinical information was extracted. Primary predictor variables were CEA and Ca 19.9. Levels of CA 19-9 and CEA were collected as continuous variables and were analyzed as binary variables: normal vs. high, using the maximum normal value as a cut-off (normal Ca 19-9 <35 U/ml; CEA <3 ng/ml). The primary outcome was overall survival from the date of diagnosis.

### Statistical analysis

Categorical variables were compared using Pearson’s Chi-squared tests and Fisher’s exact tests. Continuous variables were compared using Student *t*-tests. Survival analysis was conducted using Kaplan Meier curves, log-rank test and Cox proportional hazards model. A 95% Confidence Interval and a *p*-value of 0.05 were used to determine statistical significance. Statistical analysis was performed with SPSS 28.0.1.0 (142) (IBM Corp, Armonk, NY, USA).

## CONCLUSIONS

In summary, CA 19-9 and, to a lesser extent, CEA, show promise as prognostic markers in DA. Given the rarity of DA and the scarcity of prior research into this disease, these findings are clinically relevant to oncologists and patients alike, though larger studies are needed to validate their use and to evaluate their performance as markers of recurrence.

## Abbreviations

DA: duodenal adenocarcinoma
CEA: carcinoembryonic antigen
CA: carbohydrate antigen
