# Addendum: Importance of carbohydrate antigen (CA 19-9) and carcinoembrionic antigen (CEA) in the prognosis of patients with duodenal adenocarcinoma: a retrospective single-institution cohort study

**DOI:** 10.18632/oncotarget.28491

**Published:** 2023-12-12

**Authors:** Ellery Altshuler, Raymond Richhart, William King, Mahmoud Aryan, Akash Mathavan, Akshay Mathavan, Keegan Hones, Daniel F. Leach, Logan Pucci, Joshua Riklan, Pat Haley, Ilyas Sahin, Brian Ramnaraign, Sherise Rogers, Ibrahim Nassour, Steven Hughes, Thomas J. George, Jesus Fabregas

**Affiliations:** ^1^Department of Internal Medicine, University of Florida, Gainesville, FL 32610, USA; ^2^Department of Internal Medicine, University of Alabama at Birmingham, Birmingham, AL 35294, USA; ^3^University of Florida College of Medicine, Gainesville, FL 32610, USA; ^4^Division of Hematology and Oncology, Department of Medicine, University of Florida, Gainesville, FL 32610, USA; ^5^University of Florida Health Cancer Center, Gainesville, FL 32610, USA; ^6^Division of Surgical Oncology, Department of Surgery, University of Florida, Gainesville, FL 32610, USA; ^7^Department of Radiation Oncology, University of Florida Health, Gainesville, FL 32601, USA

**This article has an addendum:** No funding was used for this publication.

Original article: Oncotarget. 2023; 14:351–357. 351-357. https://doi.org/10.18632/oncotarget.28406

